# Rural-urban and gender differences in the association between community care services and elderly individuals’ mental health: a case from Shaanxi Province, China

**DOI:** 10.1186/s12913-021-06113-z

**Published:** 2021-01-30

**Authors:** Liu Yang, Lijian Wang, Xiuliang Dai

**Affiliations:** School of Public Policy and Administration, Xi’an Jiaotong University, No 28 Xianning West Road, Xi’an, 710049 Shaanxi China

**Keywords:** China, Elderly individuals, Mental health, Community care services, Population ageing

## Abstract

**Background:**

While community care services have been developing rapidly as a new way to meet the growing demands of elderly individuals in China, their health benefits are virtually unknown. Thus, the aim of this study was to examine the Chinese elderly individuals’ utilisation of community care services and its association with the mental health with comparing rural-urban and gender differences.

**Methods:**

For this 2019 cross-sectional study, 687 elderly people from 7 counties (districts) of China’s Shaanxi province were enrolled. Respondents’ mental health level was assessed using a self-reported mental health measure. Four categories of community care services utilisation were examined: daily care services, medical care services, social and recreational services and spiritual comfort services. The binary logistic regression model was used in examining the association between community care services utilisation and mental health.

**Results:**

Our results showed that there was a noted difference in mental health level between the male and female groups. Utilisation of medical care services and social and recreational services was significantly higher in the rural group than that in the urban group. Regression analysis showed that utilisation of daily care services (β = 0.809, *p* = 0.008) and social and recreational service (β = 0.526, *p* = 0.035) was significantly and positively associated with elderly individuals’ mental health level. Specifically, daily care services utilisation predicted a better mental health of the rural elderly (β = 1.051, *p* = 0.036) and the male elderly (β = 1.133, *p* = 0.053), while social and recreational services utilisation predicted a better mental health of the urban elderly (β = 0.927, *p* = 0.008) and the female elderly (β = 0.864, *p* = 0.007).

**Conclusions:**

Our findings indicated varied levels of community care services utilisation and mental health are common among the elderly people in China. Community care services utilisation has a positive, albeit selective, association with elderly individuals’ mental health. Further policies should strengthen the equitable development of high-quality community care services in urban and rural areas to improve the mental health of elderly individuals, and focus more on gender differences in terms of community care services needs.

**Supplementary Information:**

The online version contains supplementary material available at 10.1186/s12913-021-06113-z.

## Background

The World Health Organisation (WHO) has clearly identified the integral role that mental health plays in an individual’s overall wellness. Indeed, there can be no health without mental health [1]. The colossal growth in the worldwide ageing population has resulted in increasing attention being focused on the mental health of these individuals. For older individuals, ageing presents not only losses in physiological function, but also an increased threat of mental health problems [2–4]. In recent years, increasing mental health problems among this population have led to higher incidences of disability, declines in quality of life and increased mortality risk [5]. Addressing these issues has become a global challenge.

As one of the most populous countries, China is experiencing a rapid population ageing, becoming the country with the largest elderly population [6]. The latest population data from the National Bureau of Statistics indicates that the number of people over age 65 in China is estimated to be at 175.99 million, accounting for 12.57% of the country’s total population in 2019 [7]. It is predicted that the number of people over age 65 in China will increase to about 400 million by 2050, or 26.9% of the country’s population [8]. A report realized by the China Ageing Development Foundation in 2018 shows that 63% of the Chinese elderly often feel lonely and 62% of them often feel stressed or depressed [9], suggesting that the situation of the mental health problems among the Chinese elderly is not optimistic.

As early as 2016, the State Council issued *the outline for the ‘Healthy China 2030’ plan* aimed at improving population health to build a healthy China. This outline has proposed a healthy ageing strategy that noted the necessity of developing mental healthcare services for older adults and strengthening effective interventions for mental and psychosocial disabilities (e.g. Alzheimer’s disease) [10]. However, it is difficult to adequately address older adults’ mental health problems due to the limited professional medical resources [11]. According to official figures released by WHO in 2017, for per 100,000 residents in China, less than 8.75 mental health workers are available in China’s mental health system, which is far from satisfying people’s high demands for professional medical services [2]. Moreover, with the decline of the filial piety culture and the acceleration of modern industrialisation and urbanisation, most adult children—especially those from rural areas—choose to leave home for better job opportunities, leaving their elderly parents alone. This phenomenon has resulted in several problems for the ageing population, including declining living standards and increasing suicide incidence [12–15]. Therefore, it is imperative to develop effective and accessible approaches to address the mental health problems of China’s elderly population.

Given the decline in family support and insufficient medical and endowment resources, the Chinese government has been working to develop the community care services to promote the health and well-being of elderly individuals [16]. These services provided by local communities are aimed to satisfy various care service needs in terms of daily life support, healthcare, recreational and spiritual needs of the Chinese elderly living in the home.

Studies on the relationship between community care services and older adults’ mental health were mainly conducted in developed countries and regions, with mixed conclusions. Jacob et al. (2007) showed significant improvements in the physical and mental health and quality of life of elderly individuals who participated in community-based care programs [17]. Ormsby et al. (2010) suggested that provision of community-based men’s shed programmes, which is an activity option in the community, gives more opportunities for older men to demonstrate competence, and maintain their social networks, thus contributing positively to their physical, mental, social and occupational health [18]. Naoko (2009) revealed that consistent with stress-buffering or stress-moderating models of formal support, home and community-based services seemed to be effective for mental health among seniors with consistently low or rapidly declining daily functions [19]. Rodriguez-Romero (2020) concluded that by empowering the elderly and increasing their social support, a community intervention could help the lonely older persons stop feeling lonely and improve their health status [20]. Djernes (2006) documented low utilisation of in-home and community-based programmes among the ageing adults, noting that such social support of low quality often increase depression and exacerbate medical symptoms among the elderly [21]. Similarly, Chen and Hao (2020) found that providing basic care services, like housekeeping and visiting medical service, for ageing adults had a significant positive effect on their mental health, while providing psychological health services like spiritual comfort and psychological counselling had no such effect. This is because the quality and professional level of these services provided by communities was far from meeting ageing adults’ mental demands and promoting their mental health [22].

However, while the development of community care services in China has achieved some progress, very few studies have paid attention to the development of these services, and the association between using these services and older adults’ mental health is virtually unknown.

Limited research has been undertaken to evaluate the effects of community care services on Chinese seniors’ health, yet only the provision of community care services was examined and the health outcomes of using these services is still unclear [23]. Existing studies only focused on the rural elderly or the urban elderly in the developed regions in China [24]. Considering the separate urban-rural structure and regional economic disparity of China, it is necessary to compare the community care services utilisation, mental health level of the elderly and their associations in the specific rural and urban contexts in the moderately developed regions to improve the validity of findings. Additionally, numerous studies have showed that there were remarkable gender differences in terms of mental health level and social support [25, 26], while few studies have examined whether there exist gender differences in the utilisation of community care services and their association with the mental health of older people. Finally, given the fact that the development of community care services in China serves as a supplement to family support, it’s surprising that few studies had considered family support as potential confounding factors, and therefore, calls for further examination [27].

To fill these research gaps, firstly, the current study analysed community care services utilisation, mental health level of elderly individuals and their associations in China’s Shaanxi province, which is a moderately developed province in China. Secondly, considering rural-urban disparity and male-female difference, the results in this study were compared across gender and location groups. Finally, to reduce biases associated with omitted family support variables, instrumental support and emotional support provided by family members were controlled in this study. These findings could help Chinese policy makers understand gaps in community care services development and the association between using such services and the mental health of different groups of elderly people, thus developing strategies to improve these services.

## Methods

### Study design and sampling

The data used in this study were obtained through a cross-sectional survey conducted from June to August 2019 **[see** Additional file 1**]** [28]. This study used a stratified sampling method. The survey was conducted in Baoji, Yan’an, and Hanzhong, the pilot cities of home and community care services and ranked as moderate in economic development in Shaanxi province and in China, representing the average level. On this basis, we selected seven counties (districts) and further selected three or four typical communities from each county (district). Each of these communities had community-integrated service facilities that provide care services for the local elderly population.

### Participants

Participants taking part in this survey were elderly individuals who were 60 years old and above. During the random sampling process, elderly people with sever cognitive impairment, hearing disorder or other disabilities were excluded as they were unable to give their answers clearly. In addition, the participation was anonymous and voluntary and only the elderly who were willing to participate in were enrolled.

### Data collection

This study was approved by the medical ethics committee of Health Science Center of Xi’an Jiaotong University (approval number 2016–416). The ethics committee approves the procedure for the verbal consent, which is allowed for social investigation not involving any biological or medical experiment. Before the investigation, each elderly resident was informed of the details and the purpose of the study. Only when the respondents confirmed that they were willing to participate in this survey and gave their verbal consent, our investigators began the investigation. The survey team was composed of more than 20 members, including professors and students. For each participant in this survey, two investigators helped him/her finish the questionnaire about the personal sociodemographic characteristics, community care services utilization and mental health level. One investigator explained the question to the participant as some old adults were illiterate or unable to understand the questions and another investigator filled out the questionnaire on the base of the respondent’ answers. After filtering the data, we obtained 687 valid samples.

### Measurements

#### Dependent variable

##### Mental health level

The researchers share the WHO’s view that mental health is more than just the absence of mental disorders or disabilities, but a state of well-being in which an individual realises his or her own abilities, can cope with the normal life stresses, can work productively and is able to contribute to his or her community [1]. Therefore, in terms of the assessment of mental health in this study, the self-rated mental health was assessed using the question “In general, how do you feel about your mental state?” with possible answers including (1) very bad, (2) bad, (3) fair, (4) good and (5) very good. Self-reported health is a common and important indicator that can comprehensively and directly reflect individuals’ self-perception of their health [29, 30]. For the purposes of this study, the researchers constructed the following binary indicator for mental health in which good = 1 (based on responses of *very good* and *good*) and poor = 0 (based on responses of *fair*, *poor* and *very poor*). This method has been applied in other studies examining similar variables [31, 32].

#### Independent variables

##### Community care services utilisation

In this study, utilisation of four categories of community care services was used as the independent variables. First, 22 specific services were identified from a preliminary survey of available community care services. Second, considering collinearity problems, similar services were aggregated into four community care services categories: daily care, medical care, social and recreational and spiritual comfort services [33, 34]. Daily care services refer to some basic living support services provided by the government, neighbourhood/village committees and communities, such as housekeeping, grocery delivery and community canteen services. Medical care services refer to some basic health-related services for the elderly provided by the government, community health centres or local clinics, such as health lectures, regular medical examinations and visiting medical services. Social and recreational services refer to entertainment and cultural activities supported by the community, such as some interest groups, recreation centres and chess and card clubs. Spiritual comfort services mainly refer to services concerning and consoling elderly people’s spiritual needs, like psychological counselling and matrimonial services provided by the community. Third, we examined the sample’s community care services utilisation for each of the four categories. Specifically, a binary variable was used to indicate utilisation of each service (1 = yes; 0 = no). When a respondent reported using one or more specific services, the corresponding service category was defined as 1. If no service was used, the corresponding category was defined as 0.

##### Control variables

The demographic variables of age, sex, marital status, chronic diseases and activities of daily living (ADL) limitations were considered in this study. Household income, location (household registration), public health insurance, old-age insurance and education were included as socioeconomic status variables. Moreover, the potential effect of family support was also considered. To achieve this, family support was divided into two categories (*instrumental support* and *emotional support*) and measured by asking respondents whether they got sufficient economic support and daily care from family members and communicated frequently with family members. Education was set as a categorical variable, age as a continuous variable and the other variables as binary variables. A description of these control variables is shown in Table 1.

**Table 1:** Definition/codes of control variables

Variable	Codes/definition
Age	Continuous variable
Sex	0 = Male; 1 = Female
Marital status	0 = Non-single; 1 = Single (contains unmarried, divorced and widowed)
Household income	0 = Below-average incomes; 1 = Above-average incomes
Location	0 = Urban; 1 = Rural
Education	Categorical variable (Primary school and lower; Junior middle school; Senior middle school and higher)
Having any ADL limitation	0 = No; 1 = Yes
Having any chronic disease	0 = No; 1 = Yes
Instrumental support	0 = No; 1 = Yes
Emotional support	0 = No; 1 = Yes
Health insurance	0 = No; 1 = Yes
Old-age insurance	0 = No; 1 = Yes

Note: average income: the mean value of the total annual household income of 687 respondents

### Statistics analysis

Descriptive analysis was used to present characteristics of control variables. Chi-square analysis was used to examine whether there were statistically significant differences in community care services utilisation and mental health level and their relationships of the elderly among urban-rural groups and male-female groups. Difference examination was conducted using SPSS (Version22.0 for Windows, IBM, New York, NY, USA) statistical software. Statistical significance was set at *P* < 0.05.

To evaluate the association between community care services utilisation and the mental health level of the elderly, a binary logistic regression analysis model was implemented. To ensure the reliability of the results, we further conducted the robustness test. There are various ways to test the robustness, such as changing the testing model, changing dependent variables or independent variables and adding or removing control variables. In this study, we changed the testing model and the linear regression model was used to further examine the association between community care services utilisation and the mental health level of the elderly. All regression analyses were conducted in STATA version 15.1, and statistical significance was considered at the alpha level of *p* < 0.1, *p* < 0.05 and *p* < 0.01.

## Results

### Descriptive characteristics

Table 2 shows the respondents’ sociodemographic characteristics. The sample of 687 valid surveys—completed by elderly people in Shaanxi province with access to community care services—included 266 males and 421 females. The average respondent age was 70.42 years, with a range of 60 to 92 years. The sample included 439 (63.90%) non-single and 248 (36.10%) single individuals (i.e. those who were unmarried, divorced or widowed). Of the respondents, 344 (50.07%) had above-average household finances and 343 (49.93%) had below-average household finances. Regarding residential area, 390 (56.77%) respondents lived in urban areas and 297 (43.23%) in rural areas. Regarding education, the largest proportion of respondents (43.48%) had primary school or lower education, followed by middle school graduates (28.53%) and those with senior middle school or above education levels (28.09%). With respect to ADL limitations and chronic diseases, 16 (2.33%) respondents were determined to have an ADL limitation and 385 (56.04%) had at least one chronic disease. In terms of family support, 586 (85.30%) respondents reported receiving sufficient economic support and daily care from family members, and 583 (84.86%) communicated frequently with family members. Regarding insurance, 661 (96.22%) had old-age insurance and 673 (97.96%) had health insurance.

**Table 2:** Descriptive statistics of control variables (*N* = 687)

Variable		Number	Percentage (%)
Age	(mean, SD)	(70.42, 7.10)	–
Sex	Male	266	38.72
	Female	421	61.28
Marital status	Non-single	439	63.90
	Single ^a^	248	36.10
Household income	Above-average incomes	344	50.07
	Below-average incomes	343	49.93
Location	Urban	390	56.77
	Rural	297	43.23
Education	Primary school and lower	298	43.38
	Middle school	196	28.53
	Senior middle school and higher	193	28.09
Having ADL limitation	Yes	16	2.33
	No	671	97.67
Having any chronic disease	Yes	385	56.04
	No	302	43.96
Instrumental support	Yes	586	85.30
	No	101	14.70
Emotional support	Yes	583	84.86
	No	104	15.14
Health insurance	Yes	673	97.96
	No	14	2.04
Old-age insurance	Yes	661	96.22
	No	26	3.78

Note: Single ^a^ contains unmarried, divorced and widowed

### Mental health and community care services utilisation for different groups

**Fig. 1:**
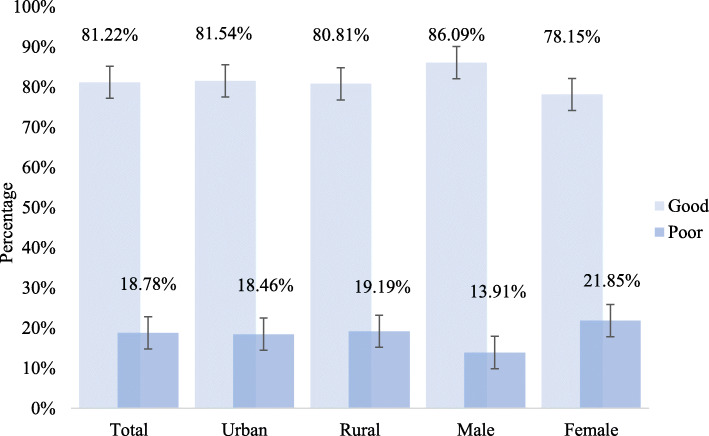
Mental health level of older individuals

While there was no significant difference in mental health level between the urban and rural groups, there was a noted difference between the male and female groups (see Fig. 1**[**see Figure file 1**]**). The percentage of urban respondents with good mental health (81.54%) was only slightly higher than that of the rural respondents (80.81%). In contrast, the percentage of older men who claim having a poor mental health (13.91%) is significantly lower than that of older women (21.85%) (*p* = 0.009).

Table 3 shows a comparison of community care services utilisation between urban-rural and male-female groups. Data showed that of the 687 respondents, 209 (30.42%) used at least one daily care service, while 478 (69.58%) never used any daily care service. The daily care services utilisation of urban and male older adults was slightly more frequent than those of the rural and female older adults. In medical care services utilisation, 498 (72.49%) respondents reported using at least one medical care service, while 189 (27.51%) had never used such service. Medical care services utilisation varied by location, with older people living in rural areas more likely to use medical care services than those living in urban areas (*p* = 0.043). Of the respondents, 432 (62.88%) reported that they used at least one social and recreational service and 255 (37.12%) never used any of such service. Residence area was found to affect social and recreational services utilisation, with 67.34% of rural ageing individuals using these services, indicating a significantly higher rate than that of those living in urban areas (*p* = 0.035). Regarding spiritual comfort services, 131 (19.07%) respondents used at least one spiritual comfort service, while 556 (80.93%) never used any of these services. Of the respondents who used spiritual comfort services, 82 (21.03%) were urban residents and 56 (21.05%) were male.

**Table 3:** Community care services utilisation of older individuals among different groups

Categories of Community Care Services	Total (*N* = 687)	Urban (*N* = 390)	Rural (*N* = 297)	*P* values	Male (*N* = 266)	Female (*N* = 421)	*P* values
Daily care services (n, %)				0.370			0.855
Yes	209 (30.42)	124 (31.79)	85 (28.62)		82 (30.83)	127 (30.17)	
No	478 (69.58)	266 (68.21)	212 (71.38)		184 (69.17)	294 (69.83)	
Medical care services (n, %)				0.043 *			0.278
Yes	498 (72.49)	271 (69.49)	227 (76.43)		199 (74.81)	299 (71.02)	
No	189 (27.51)	119 (30.51)	70 (23.57)		67 (25.19)	122 (28.98)	
Social and recreational services (n, %)				0.035 *			0.275
Yes	432 (62.88)	232 (59.49)	200 (67.34)		174 (65.41)	258 (61.28)	
No	255 (37.12)	158 (40.51)	97 (32.66)		92 (34.59)	163 (38.72)	
Spiritual comfort services (n, %)				0.135			0.293
Yes	131 (19.07)	82 (21.03)	49 (16.50)		56 (21.05)	75 (17.81)	
No	556 (80.93)	308 (78.97)	248 (83.50)		210 (78.95)	346 (82.19)	

Note: ^*^p < 0.05

### Multivariate regression results

Table 4**(placed at the end of the document)** shows the multivariable regression results of the binary logistic regression models. The results of model 1 showed that daily care services (β = 0.809, *p* = 0.008) and social and recreational services (β = 0.526, *p* = 0.035) were significantly and positively associated with mental health level of the 687 elderly individuals. The associations between mental health level and medical care services and spiritual comfort services were positive, but not significant.

**Table 4:** The association between community care services utilisation and the mental health level of older individuals

Variables	Model 1	Model 2	Model 3	Model 4	Model 5
	Total	Urban	Rural	Male	Female
Community care services utilisation (ref: no)					
Daily care services	0.809 (0.307) ***	0.721 (0.434)	1.051 (0.501) **	1.133 (0.586) *	0.550 (0.385)
Medical care services	0.106 (0.269)	0.029 (0.344)	0.243 (0.496)	0.146 (0.546)	0.024 (0.325)
Social and recreational services	0.526 (0.250) **	0.927 (0.352) ***	0.306 (0.413)	−0.210 (0.455)	0.864 (0.318) ***
Spiritual comfort services	0.217 (0.349)	0.761 (0.506)	−0.437 (0.554)	0.308 (0.563)	0.198 (0.473)
Instrumental support (ref: no)	0.984 (0.336) ***	0.424 (0.508)	1.567 (0.522) ***	1.289 (0.612) **	0.784 (0.443) *
Emotional support (ref: no)	0.885 (0.334) ***	1.313 (0.504) ***	0.632 (0.505)	0.389 (0.615)	1.204 (0.432) ***
Health insurance (ref: no)	0.700 (0.684)	0.227 (1.280)	0.974 (0.958)	3.881 (2.350)	0.461 (0.760)
Old-age insurance (ref: no)	0.600 (0.481)	0.191 (0.679)	1.153 (0.840)	−0.438 (1.192)	0.912 (0.590)
Age	0.031 (0.018) *	0.079(0.026) ***	−0.037 (0.029)	−0.020(0.032) *	0.059 (0.024) **
Sex (ref: male)	−0.117 (0.268)	0.131 (0.367)	−0.911 (0.453) **	–	–
Marital status (ref: non-single)	−0.239 (0.252)	− 0.402 (0.348)	−0.016 (0.418)	− 0.346 (0.483)	−0.166 (0.310)
Household income (ref: below-average incomes)	1.639 (0.274) ***	1.521 (0.364) ***	1.850 (0.453) ***	2.195 (0.534) ***	1.570 (0.341) ***
Location (ref: urban areas)	0.353 (0.256)	–	–	1.143 (0.539) **	0.108 (0.317)
Education (ref: primary school and lower)					
Junior middle school	0.756 (0.356) **	0.775 (0.429) *	2.251 (1.144) **	0.344 (0.572)	1.529 (0.594) **
Senior middle school and higher	0.491 (0.348)	0.287 (0.407)	2.531 (1.177) **	−0.254 (0.541)	1.642 (0.598) ***
Having any ADL limitation (ref: no)	−2.750 (0.662) ***	−3.385 (0.966) ***	−1.835 (0.965) *	−2.552 (0.920) ***	−3.320 (0.990) ***
Having any chronic disease (ref: no)	−0.608 (0.244) **	−0.245 (0.326)	−1.431 (0.431) ***	− 0.800 (0.462) *	−0.670 (0.309) **
Observation	687	390	297	266	421
Pseudo R^2^	0.259	0.281	0.33	0.252	0.252

Note: The table contains the β coefficient (standard error) from logit models. ^*^p < 0.1. ^**^p < 0.05. ^***^p < 0.01

Some sociodemographic characteristics were also significantly related to mental health level of the respondents. The elderly reported a better mental health as they got older (β = 0.031, *p* = 0.096). Additionally, respondents who graduated from junior middle school reported having a better mental health than those with a primary school degree or lower (β = 0.756, *p* = 0.034). Compared with those with lower household incomes, respondents with higher incomes reported having a better mental health (β = 1.639, *p* = 0.000). As expected, having no ADL limitations or chronic diseases also predicted a better mental health (β = − 2.750, p = 0.000; β = − 0.608, *p* = 0.013). In terms of family support, getting sufficient economic support and daily care and communicating frequently with family were significantly associated with a better mental health (β = 0.984, *p* = 0.003; β = 0.885, *p* = 0.008).

The results of Model 2 to Model 5 showed that the associations between mental health level and community care services utilisation varied between urban-rural groups and male-female groups. Specifically, using daily care services utilisation had a significantly positive relationship with the mental health level of male and rural respondents (β = 1.133, *p* = 0.053; β = 1.051, *p* = 0.036), while using social and recreational services was positively and significantly associated with the mental health level of female and urban respondents (β = 0.864, *p* = 0.007; β = 0.927, *p* = 0.008).

### Robustness test results

Table 5 shows the multivariable regression results of the linear regression models. To ensure the reliability of the results in Table 4, we further changed the testing model to test the robustness of the results. The dependent variable (mental health) was set as a continuous variable (ranging from 1 to 5) and the associations between community care services utilisation and mental health level of older individuals were examined in the linear regression models. The results showed that the significance and direction of the coefficient of dependent variables in Model 6 to model 10 were consistent with that in Model 1 to model 5, which suggested that the estimates and results of the association between dependent and independent variables in this study were robust and reliable.

**Table 5:** Robustness test: multivariable regression results of the linear regression models

Variables	Model 6	Model 7	Model 8	Model 9	Model 10
	Total	Urban	Rural	Male	Female
Community care services utilization (ref: no)					
Daily care services	0.149 (0.071) **	0.098 (0.094)	0.226 (0.112) **	0.214 (0.106) **	0.109 (0.094)
Medical care services	0.045 (0.071)	0.021 (0.089)	0.065 (0.122)	0.035 (0.112)	0.049 (0.092)
Social and recreational services	0.135 (0.068) **	0.257 (0.088) ***	0.020 (0.109)	−0.032 (0.102)	0.188 (0.090) **
Spiritual comfort services	0.080 (0.082)	0.070 (0.106)	−0.086 (0.131)	0.079 (0.116)	0.103 (0.114)
CV	Yes	Yes	Yes	Yes	Yes
Observation	687	390	297	266	421
Adj R2	0.213	0.229	0.213	0.16	0.252

Note: CV: control variables; The table contains the β coefficient (standard error) from logistic models and regression models. *p < 0.1. **p < 0.05. ***p < 0.01

## Discussion

Survey data collected in the Shaanxi province in 2019 were used to examine the associations between four types of community care services utilisation and the mental health level of older individuals, including the differences of these associations among urban-rural and male-female groups. Data analysis revealed three crucial findings. First, the mental health level of China’s older population is not optimal, especially among women. Second, there were some differences and inequities in community care services utilisation among the elderly in urban and rural areas. Third, while the use of daily care services and social and recreational services was positively related to the mental health level of these respondents, the differences of the relationship were observed in rural-urban groups and male-female groups.

The results of this study showed that 18.78% of the respondents reported having a poor mental health. This finding highlights the unoptimistic situation of the mental health problems among China’s ageing citizens. Additionally, the mental health of older women was found to be significantly worse than that of older men, a finding consistent with those of other studies [35]. In another finding, while the mental health of urban older individuals was slightly better than that of those living in rural areas, the difference was not significant. Although the overall mental health of all of China’s ageing population is of concern, these results suggested that more attention should be focused on meeting the pressing needs of the rural and female elderly populations.

Interestingly, medical care services utilisation was significantly higher in rural areas than in urban areas. In fact, the unequal development of public health resources and services between urban and rural areas has been a long-standing problem in China [36]. As an important part of the Chinese healthcare system, the development of community care services is worse in terms of both quality and quantity in rural areas, especially poorer areas [37]. According to official data released by the Ministry of Civil Affairs in 2018, the coverage rate of community-integrated service facilities in urban areas was 78.7%, but only 45.3% in rural areas [38]. However, due to the deficiency of large hospitals and restricted transportation, many rural residents have fewer chances to access professional medical resources in bigger medical institutions compared with urban areas. Therefore, the elderly in rural areas might turn to community health centres or local clinics for some basic treatments as they have health problems, while those in urban areas are prone to get some professional and advanced medical treatments from large hospitals [39]. This might explain the unexpected finding that although there are fewer and worse healthcare facilities and healthcare services in rural areas, the rural participants use medical care services provided by communities more frequently than urban participants.

Our results showed that the proportion of the elderly population accessing social and recreational services in rural areas is significantly higher than those in urban areas. This finding seemed to be inconsistent with some existing studies which suggested that rural older people were less socially active than urban older people [40]. A possible explanation for this is that apart from social and recreational services provided by the community, urban older people have plenty of choices of social activities because economy, public transportation and entertainment facilities and services are considerably more developed and accessible than are those in rural areas [41]. While rural older people have relatively limited entertainment ways, thus they might use local community social and recreational services more frequently than urban older people. Furthermore, due to the accelerative urbanization and industrialization in China, more and more adult children are flowing from rural areas to urban and developed areas, leading to the increase of ‘empty nesters’ (the elderly who live alone or only live with their spouse without support from children) in rural China [42]. In this instance, some of the rural elderly prefer to seek new channels, such as participating in community social activities, to extend their social network and social support.

Key findings of this study indicated that after controlling for sociodemographic variables, the utilisation of daily care services and social and recreational services was positively associated with the mental health level among older adults. To be specific, older people who receive daily care services had a higher probability of having good mental health. The probable reason for this finding is the fact that individuals receiving daily care from community care centre staff likely feel supported and secure, which benefits their mental health [19, 43–45]. Furthermore, receiving professionally provided services frees older individuals from the burden of housework, allowing them to enjoy more leisure time activities that can boost their mood [23].

The results among rural and urban respondents had key differences. For example, while the utilisation of daily care services predicted better mental health among elderly individuals in rural areas, this association was not observed in urban areas. Applying Maslow’s hierarchy of needs theory, the underlying reason for this finding might be attributed to the fact that, compared with those living in urban areas, the care service needs of older individuals living in rural areas are more basic [46]. Furthermore, because rural residents highly value the tradition of hard work, they are more likely to continue working as they aged, as long as their physical condition allows. In this case, receiving daily care services (e.g. meal delivery, housekeeping and grocery delivery services) could reduce their labour burden to a large extent, which may contribute to family well-being [24].

In terms of male-female differences, the positive association between daily care services utilisation and the mental health level was significant only among the male elderly. With the traditional roles of men breadwinners and women homemakers, females generally have played the primary role of taking care of families [47, 48]. Under the influence of the traditional gender role attitude, women might feel lost when they get older and shift into the role of receiving such services instead of providing them [49, 50]. Therefore, older women’s possible feelings of worthlessness from getting daily care services from others may offset the potential benefit of relieving them from the burden of housework. This phenomenon may help explain the result that no significant positive association was observed in the female group from receiving such services.

Similar to findings presented in prior studies, the results of this study indicated that using social and recreational services was positively and significantly correlated with older adults’ mental health level [51, 52]. According to the activity theory, participating in diverse social activities can refresh and improve self-cognition of ageing adults and reduce their sense of isolation from the ever-changing society around them. Therefore, it is beneficial for older adults to expand their social networks and obtain a variety of health-related information [53]. Additionally, community social and recreational services create new opportunities for the elderly to get together with kindred spirits, which could help make up for deficiencies in interpersonal interactions with their family members and satisfy their demand for a better life [54].

Regarding rural-urban differences, social and recreational services utilisation predicted better mental health among urban residents, but not rural residents. The researchers speculated that despite some progress in promoting the development of elderly services in rural areas, significant gaps remain between urban and rural service content and quality due to restrictions related to economic and policy factors [55]. In some cases, lower level of community care services and activities (e.g. mahjong, a prevailing gambling game in rural China) may not help increase positive health behaviours among the rural elderly [21, 56, 57]. In addition, although social and recreational services utilisation was higher among the rural residents, the social networks and social capital they accessed through these services were limited. In contrast, the urban elderly may be more likely to access beneficial health-related information and resources by engaging in community activities and broadening their social circles, thus significantly promoting their psychological well-being [58].

Regarding male-female differences, the utilisation of social and recreational services had a positive and significant relationship with women’s mental health, but not with men’s. The probable reason for this finding may be that, women, compared with men, spend more time in providing and receiving social support and have more extensive and better social relations. Therefore, they would experience more benefits in health terms from social activities than men do [59]. Moreover, due to gender gaps in family labour division and life habits, elderly women tend to shoulder more household chores. In such cases, participating in community activities can improve elderly women’s living environments to a much greater extent, making their mental health more sensitive to these social activities than elderly men [60].

One unexpected finding was that utilisation of medical care services and spiritual comfort services was not significantly correlated with the mental health of the individuals in our study. Due the fact that the establishment of community care services in China is still in its infancy, the lack of qualified professionals and facilities constrains the provision and utilisation of diverse and high-quality care services for older adults, especially community medical and psychological care [61]. Therefore, the poor accessibility and low quality may contribute to the result that utilisation of these services did not have a significant correlation with the respondents’ mental health level [22].

We also noticed that getting sufficient economic support and daily care from family members and communicating frequently with family members were positively and significantly associated with good mental health of older adults. This finding indicated that family support, including instrumental and emotional support, still plays an essential and central role in elderly care in China [62]. In contrast, public health and old-age insurance did not show a significant association with the respondents’ mental health level. As expected, as an important component of formal social support, adequate and accessible community care services have a more positive relationship with the health of older adults compared with social insurance with extensive coverage [63].

These findings also suggested that multi-generation cohabitation should be encouraged to evoke children’s sense of family responsibility and willingness to provide support for their elderly family members. The government and local communities also should work together to support the construction of old-age facilities and provide more and higher-quality services for the ageing population. Specifically, efforts should be focused on the provision of daily care and social and recreational services. Medical care and spiritual comfort services should also be strengthened to fully meet the needs and improve the health of community-dwelling older people. During this process, it is essential to implement effective policy measures to promote the equitable development of these services in both urban and rural areas. Furthermore, to enhance their mental health, more attention should focus on the community care services preferences of different population groups in China, especially those of older women.

### Strength and limitations

This study is one of the very few that have examined the association between community care services utilisation and mental health level among China’s ageing population using cross-sectional survey data. This study also considered disparities and discrepancies between male-female and rural-urban groups, an approach not found in previous studies. Our results confirmed that the utilisation of daily care services and social and recreational services was associated with better mental health of older people in China. The results also revealed the significant male-female discrepancies and rural-urban disparities in community care services utilisation, mental health level and associations between these variables among a sample of ageing adults. These findings could help inform the development and implementation of more targeted and effective community care services strategies.

Despite the value of these findings, this study did have some limitations that need to be considered. First, the association between community care services utilisation and respondents’ mental health level was assessed used the binary logistic regression model with a cross-sectional survey. Therefore, the possible time lag between services utilisation and mental health outcomes was not considered and the exact causal relationship still needs further investigation. Second, the survey was conducted only in three cities of the Shaanxi province, representing an intermediate economy level in China. More regions, such as Xi’an, which has one-third of Shaanxi’s population and represents the well-developed economy level in China, may be included in future studies to explore the differences between regions having different economic levels. Third, mental health of the older population is a complex issue. Although some potential variables were controlled for in this study, there might be unmeasurable variables that were ignored, resulting bias in the results. Finally, while we tried our best to strengthen the normativity and scientificity of the survey process, there might be some social desirability bias in this study.

## Conclusions

In summary, this study draws three conclusions. First, rural and female older individuals suffered worse mental health, particularly older women. Second, community care services utilisation was unbalanced among urban-rural groups. Third, community care services had a positive, albeit selective, association with the mental health level of older adults in China. Specifically, daily care services and social and recreational services were positively associated with good mental health. Furthermore, daily care services utilisation predicted a better mental health of older men and older people living in rural areas, while social and recreational services utilisation predicted a better mental health of older women and older people living in urban areas. The findings of this study provided some important insights on how to develop more effective community care services for the ageing population in China.

## Supplementary Information

**Additional file 1.**

## Data Availability

The datasets used and/or analyzed in this study belong to our research team, and data does not involve any personal privacy information. The datasets are available from the corresponding author on reasonable request.

## Abbreviations

WHO: The World Health Organisation
ADL: Activities of daily living
