# Supplementary material for: Rural-urban and gender differences in the association between community care services and elderly individuals’ mental health: a case from Shaanxi Province, China
Source: BMC Health Serv Res. 2021 Jan 30;21:106. doi: 10.1186/s12913-021-06113-z (PMC7847576; doi:10.1186/s12913-021-06113-z)
Supplement: Supplementary file 1 — Additional file 1. [file 12913_2021_6113_MOESM1_ESM.docx]

# The High-quality Development of China’s Undertakings for The Aged Study**

____City ____County / district ____Street / township ____Community / old-age care institution

Dear Sir / Madam:

We are the research group of Xi'an Jiaotong University. According to the research plan of “The High-quality Development of China’s Undertakings for The Aged Study”, we conduct this survey to investigate and understand your opinions on the development of aging cause and industry. We promise that the survey will be anonymised, and the data will be for research only. Please answer these questions truthfully. Thank you for your participation and support!

**Part 1: Basic Information**

| **Serial Number** | **Items** | **Options** |
| --- | --- | --- |
| 1.1 | Age | ______ years old |
| 1.2 | Sex | 1) Male; 2) Female |
| 1.3 | Education Level | 1) Primary school and lower; 2) Junior middle school; 3) Senior middle school and higher |
| 1.4 | Marital Status | 1) Unmarried; 2) Married; 3) Divorced; 4) Widowed |
| 1.5 | Hukou Location | 1) Urban; 2) Rural |
| 1.6 | Household Income | Total income in 2018 ________yuan |
| 1.7 | Household Expenditure | Total expenditure in 2018 ________yuan |
| 1.8 | Living Mode | 1) Living in the home and community; 2) Living in old-age care institution |
| 1.9 | Primary Care Personnel (Multiple Choices) | 1) In person; 2) Spouse; 3) Children; 4) Nurse; 5) Community Service Worker;6) others ______ |
| 1.10 | Number of Children | Total number: ______  Number (son): _____  Number (daughter): _____ |
| **1.11 Evaluation on health and living Condition** | | |
| Which of the following activities of daily living (ADL) cannot be finished by yourself? (Multiple Choices) | | 1) Eating; 2) Dressing; 3) Getting in and out bed; 4) Going to the toilet; 5) Taking a bath; 6) Walking on flat ground; 7) Walking up and down stairs; 8) None |
| Which of the following chronic diseases do you have? (Multiple Choices) | | 1) Cardiovascular and cerebrovascular diseases (e.g. coronary heart disease, cerebral infarction and hypertension); 2) Osteoarthropathy; 3) Chronic bronchitis; 4) Chronic gastroenteritis; 5) Diabetes; 6) Sequela of cerebrovascular accident; 7) others ______; 8) None |
| How has your chronic diseases changed in last year? | | 1) Worse; 2) Unchanged; 3) Better |
| How do think about your physical health? | | 1) Very poor; 2) Poor; 3) Fair; 4) Good; 5) Very Good |
| How do think about your mental health? | | 1) Very poor; 2) Poor; 3) Fair; 4) Good; 5) Very Good |
| Taking all things together, how satisfied are you with your life these days? | | 1) Very dissatisfied; 2) dissatisfied; 3) Fair; 4) Satisfied; 5) Very satisfied |
| How do think about the relationship with your family members? | | 1) Very bad; 2) Bad; 3) Fair; 4) Good; 5) Very Good |
| Do you get sufficient economic support and daily care from your family members? | | 1) Very agree; 2) Agree; 3) Fair; 4) Disagree; 5) Very disagree |
| Do you communicate frequently with your family members | | 1) Very agree; 2) Agree; 3) Fair; 4) Disagree; 5) Very disagree |
| Are you used to the living environment in the community / old-age care institution： | | 1) Very agree; 2) Agree; 3) Fair; 4) Disagree; 5) Very disagree |

**Part 2: Pension System**

| **Serial Number** | **Items** | **Options** |
| --- | --- | --- |
| 2.1 | Which of the following old-age insurance do you have? (Multiple Choices) | 1) Statutory old-age insurance for urban workers; 2) Pension for government and public institution employees; 3) Basic old-age insurance for urban and rural residents; 4) Commercial endowment insurance; 5) Others_____; 6) None |
| **2.2 Evaluations on old-age insurance** | | |
| Do you feel pressure to pay for your old-age insurance? | | 1) Very agree; 2) Agree; 3) Fair; 4) Disagree; 5) Very disagree |
| How satisfied are you with the standard of treatment of insurance? | | 1) Very dissatisfied; 2) dissatisfied; 3) Fair; 4) Satisfied; 5) Very satisfied |
| 2.3 | Which of the following health insurance do you have? (Multiple Choices) | 1) Medical insurance for urban employees; 2) Medical insurance for urban residents; 3) The new countryside cooperative medical scheme; 4) Medical insurance for government and public institution employees; 5) The enterprise subsidiary medical insurance; 6) [Commercial health insurance](http://dict.youdao.com/w/commercial%20health%20insurance/#keyfrom=E2Ctranslation); 7) Others_____; 8) None |
| **2.4 Evaluations on health insurance** | | |
| Do you feel pressure to pay for your health insurance? | | 1) Very agree; 2) Agree; 3) Fair; 4) Disagree; 5) Very disagree |
| How satisfied are you with the standard of treatment of insurance? | | 1) Very dissatisfied; 2) dissatisfied; 3) Fair; 4) Satisfied; 5) Very satisfied |

**Part 3:** **Demand, Utilisation and Supply of Services for the Aged**

| **Serial Number** | **Items** | **Options** |
| --- | --- | --- |
| 3.1 | Do you know about the care services provided by your community / old-age care institution? | 1) Very agree; 2) Agree; 3) Fair; 4) Disagree; 5) Very disagree |
| **3.2 Daily care services** | | |
| Daily care services facilities provided by your community / old-age care institution (Multiple Choices) | | 1) Canteen; 2) Bathhouse; 3) Care services center / [day care center](http://dict.youdao.com/w/Day%20Care%20Center/#keyfrom=E2Ctranslation); 4) Commercial old-age care service company; 5) Others_____; 6) None |
| Daily care services provided by your community / old-age care institution (Multiple Choices) | | 1) Housework services;  2) Community canteen services;  3) Grocery delivery;  4) Safety-guaranteed services;  5) Maintenance services;  6) Others_____;  7) None |
| Daily care services you have used (Multiple Choices) | |  |
| Daily care services you need (Multiple Choices) | |  |
| **3.3 Medical care services** | | |
| Medical care services facilities provided by your community / old-age care institution (Multiple Choices) | | 1) Clinic; 2) Pharmacy; 3) Health service center;4) Rehabilitation treatment room; 5) Others_____; 6) None |
| Medical care services provided by your community / old-age care institution (Multiple Choices) | | 1) Health lectures;  2) Regular medical examinations;  3) Health records;  4) Visiting medical services;  5) Rehabilitation nursing services;  6) Hospice care services;  7) Online family doctor services;  8) Others_____;  9) None |
| Medical care services you have used (Multiple Choices) | |  |
| Medical care services you need (Multiple Choices) | |  |
| **3.4 Social and recreational services** | | |
| Social and recreational services facilities provided by your community / old-age care institution (Multiple Choices) | | 1) Senior Citizen Activity Center; 2) Chess room; 3) Reading room; 4) Calligraphy and painting room; 5) Fitness room; 6) Others_____; 7) None |
| Social and recreational services provided by your community / old-age care institution (Multiple Choices) | | 1) Interest groups;  2) Recreation centres;  3) Chess and card clubs;  4) Performance activities  5) Calligraphy and painting shows;  6) Knowledge lectures;  7) Online classes;  8) Others_____;  9) None |
| Social and recreational services you have used (Multiple Choices) | |  |
| Social and recreational services you need (Multiple Choices) | |  |
| **3.5 Spiritual comfort services** | | |
| Spiritual comfort services facilities provided by your community / old-age care institution (Multiple Choices) | | 1) Psychological counselling room;  2) Others_____;  3) None |
| Spiritual comfort services provided by your community / old-age care institution (Multiple Choices) | | 1) Psychological counselling services;  2) Home visiting services;  3) Matrimonial services;  4) Others_____;  5) None |
| Spiritual comfort services you have used (Multiple Choices) | |  |
| Spiritual comfort services you need (Multiple Choices) | |  |

**Part 4: Evaluation on Services for the Aged**

**Part 5: Evaluation on the Aged Productions**

Investigator ( [signature](http://dict.youdao.com/w/signature/#keyfrom=E2Ctranslation)) : ________ Date: __________

**Note: The whole questionnaire includes 157 items and five parts: basic information, pension system, demand utilisation and supply of services for the aged, evaluation on services for the aged and evaluation on the aged productions. In consideration of large amounts of items of the questionnaire and the requirements of our research group, part of the questionnaire content are listed, which are related to this study.
